# Cavity-Suppressing Electrode Integrated with Multi-Quantum Well Emitter: A Universal Approach Toward High-Performance Blue TADF Top Emission OLED

**DOI:** 10.1007/s40820-022-00802-y

**Published:** 2022-02-11

**Authors:** Il Gyu Jang, Vignesh Murugadoss, Tae Hoon Park, Kyung Rock Son, Ho Jin Lee, WanQi Ren, Min Ji Yu, Tae Geun Kim

**Affiliations:** School of Electrical Engineering, Korea University, Anam-ro 145, Seongbuk-gu, Seoul, 02841 Republic of Korea

**Keywords:** Cavity suppression, Multi-quantum well, Viewing angle, Efficiency roll-off, Top emission OLED

## Abstract

A novel device structure for thermally activated delayed fluorescence (TADF) top emission organic light-emitting diodes (TEOLEDs) that improves the viewing angle characteristics and reduces the efficiency roll-off is presented. Furthermore, we describe the design and fabrication of a cavity-suppressing electrode (CSE), Ag (12 nm)/WO_3_ (65 nm)/Ag (12 nm) that can be used as a transparent cathode. While the TADF-TEOLED fabricated using the CSE exhibits higher external quantum efficiency (EQE) and improved angular dependency than the device fabricated using the microcavity-based Ag electrode, it suffers from low color purity and severe efficiency roll-off. These drawbacks can be reduced by using an optimized multi-quantum well emissive layer (MQW EML). The CSE-based TADF-TEOLED with an MQW EML fabricated herein exhibits a high EQE (18.05%), high color purity (full width at half maximum ~ 59 nm), reduced efficiency roll-off (~ 46% at 1000 cd m^−2^), and low angular dependence. These improvements can be attributed to the synergistic effect of the CSE and MQW EML. An optimized transparent CSE improves charge injection and light outcoupling with low angular dependence, and the MQW EML effectively confines charges and excitons, thereby improving the color purity and EQE significantly. The proposed approach facilitates the optimization of multiple output characteristics of TEOLEDs for future display applications.

**Supplementary Information:**

The online version contains supplementary material available at 10.1007/s40820-022-00802-y.

## Highlights

- A blue thermally activated delayed fluorescence top-emission organic light-emitting diode (TEOLED) structure that combines a cavity-suppressing transparent electrode and a multi-quantum-well emissive layer is proposed.
- The TEOLED exhibits high external quantum efficiency (18.05%), high color purity (full width at half maximum ~ 59 nm), reduced efficiency roll-off (~ 46% at 1000 cd m^−2^), and low angular dependence

**Supplementary Information:**

The online version contains supplementary material available at 10.1007/s40820-022-00802-y.

## Graphical abstract

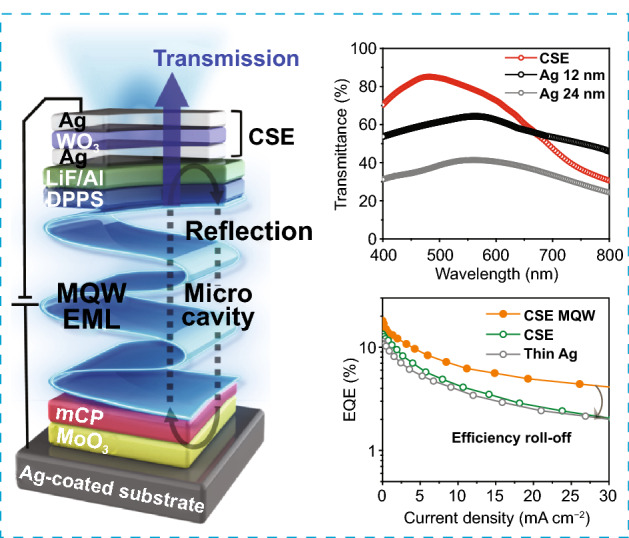

## Introduction

Research on organic light-emitting diodes (OLEDs) has made rapid progress since the first demonstration of these devices by Tang and VanSlyke [1], and it has led to their commercialization for display applications. Over the last decade, significant research efforts have been devoted toward improving the efficiency of OLEDs, which has resulted in the development of phosphorescent and thermally activated delayed fluorescent (TADF) emitters with an internal quantum efficiency (IQE) of approximately 100% [2]. Compared to bottom-emitting OLEDs (BEOLEDs), top-emitting OLEDs (TEOLEDs) are more suited to use in display technologies and biomedical applications because they facilitate the use of nontransparent backplane technology for active-matrix OLEDs [3]. In addition, TEOLEDs offer the advantages of a high aperture ratio and high image quality, which makes them potential candidates for sunlight-readable displays such as smartphones, wrist-worn smartwatches, tablets, and automotive head-up displays [4–6]. However, despite the near 100% IQE of organic emitters, efficient outcoupling remains challenging because a large amount of light is trapped inside various components owing to total internal reflection at interfaces and the surface plasmon losses/absorption of metals [7]. To overcome these challenges, optical cavity effects due to light refraction and trapping between layers with an adequately large refractive index contrast have been widely used in top-emitting devices with relatively strong internal reflections between the top and bottom electrodes [8]. Studies have demonstrated that the efficiency and color purity of TEOLEDs can be improved significantly by employing a microcavity structure without any significant modifications to the device configuration [9–11]. However, devices with a microcavity structure exhibit increased angular dependence and lower contrast ratio owing to strong reflection at high luminance levels [12–14]. Therefore, the optical cavity effect should be adequately attenuated to maximize the light extraction (or outcoupling) efficiency of TEOLEDs.

To suppress the aforementioned adverse effects of the microcavity structure, nanoporous polymer films have been used as polarizers/diffusers [13, 15]. However, these two-dimensional polymer nanostructures degrade the current efficiency of the devices owing to light scattering [16]. This problem can be solved by replacing the two-dimensional nanoporous polymer film with a three-dimensional polymer film. However, such polymer nanostructures cannot be easily integrated with TEOLEDs because of the complexity of their deposition process, which involves spin-coating or lithography [17]. Further, light scattering due to polymer thin films may additionally lead to pixel crosstalk [18].

Multilayer electrodes, such as oxide/metal/oxide (OMO) [19, 20], which do not require the use of additional films and offer high transmittance and low sheet resistance, can be utilized as top electrodes to control cavity effects in TEOLEDs. In such structures, cavity effects can be suppressed by reducing the thickness of the metal layer sandwiched between top and bottom oxide layers. In this manner, the transmittance of top electrodes can be improved. However, this approach significantly increases the sheet resistance of OMO electrodes. Similarly, a metal/organic/metal (MOM) structure, inspired by the Fabry–Pérot resonator, has been used as a cathode in BEOLEDs to improve light extraction and color purity and suppress the cavity effect [21, 22]. Unlike the OMO case, we can suppress cavity effects without sacrificing transmittance and sheet resistance by using MOM-based transparent electrodes because anti-reflective effects at the interface between the metal and oxide layers can be optimized for relatively thick metal layers [23]. Sung et al. employed an Ag/NPB/Ag structure as a cathode in transparent OLEDs and demonstrated that its efficiency varies dramatically with the thickness of the organic layer [24]. However, the low carrier density and charge mobility of organic materials limit their use as transparent electrodes.

To solve this problem, we have explored inorganic oxide materials that can replace conventional organic materials and selected an Ag/WO3/Ag structure as a cavity-suppressing transparent cathode for TADF-TEOLEDs. In this structure, we used WO_3_ as a metal oxide layer because it is known as an n-type dielectric material with a high refractive index, low optical loss in the visible region, and high electron mobility; further, it can be readily deposited by means of thermal evaporation [25, 26]. The optical transmittance of Ag/WO_3_/Ag-based cavity-suppressing electrode (CSE) can be tuned by varying the thickness of the WO_3_ layer without compromising its sheet resistance [27].

Despite the use of a transparent cathode, such as the CSE, it remains challenging to achieve high color purity, high brightness, and excellent efficiency, while significantly reducing the efficiency roll-off at high current densities in OLEDs. These features are essential for reducing energy consumption in industrial applications of OLEDs. The leading causes of efficiency roll-off in TADF-based OLEDs are triplet quenching by polarons (triplet-polaron annihilation (TPA)) and other triplets (triplet–triplet annihilation (TTA)) [28, 29]. From a materials engineering viewpoint, triplet quenching can be alleviated by using host–guest doping systems in the fabrication of TADF-based OLEDs. However, doping is a complex strategy and often lacks reproducibility, which hinders large-scale manufacturing [30]. Although a few undoped TADF-OLEDs have been reported, a practical level of efficiency has not been achieved yet. The use of multi-quantum well (MQW) emissive layers (EML), which help confine charge carriers and excitons and broaden the exciton recombination zone (ERZ), has been reported as an efficient approach to overcome these challenges and reduce efficiency roll-off [31, 32]. Li et al. [29] demonstrated a six-fold reduction in efficiency roll-off in green TADF-TEOLEDs by using an MQW structure. Compared to host–guest doping systems, the CSE MQW-EML approach appears complex because the thickness of many layers must be precisely controlled. However, the proposed approach yields a uniform film under optimal deposition conditions, and the film thickness can be tightly controlled because all processes are conducted in a single run by using a fully computerized thermal evaporator. By contrast, with host–guest doping systems, it is considerably difficult to control relative doping concentrations, and phase segregation occurs [30]. In this respect, the proposed methodology may be more reproducible than conventional doping methods, and it offers more advantages in terms of large-scale manufacturing. These results motivated us to introduce an MQW EML in CSE-based blue TADF-TEOLEDs to alleviate the challenges pertaining to color purity and efficiency roll-off.

In this study, we propose a novel device structure with a CSE and a MQW EML to realize blue TADF-TEOLEDs with multiple output characteristics. We optimize the thickness of the CSE layers based on the results of an optical simulation. The optimized CSE exhibits a low sheet resistance (≈ 2.24 Ω sq^−1^) while maintaining a high transmittance (≈ 85%) for blue emission (480 nm). Previously reported OMO-based electrodes suffer from a trade-off between transmittance and conductivity, and they have low figure of merit (FoM) values [20, 33]. However, we can increase conductivity without transmittance losses by employing the CSE (with a MOM structure), thus solving the trade-off problem, and, eventually, improving the FoM. We optimize the thickness and number of quantum wells (QWs) experimentally. The TADF-TEOLEDs fabricated using seven QWs and the optimized CSE exhibit outstanding performance with high color purity, bright luminescence, an EQE of approximately 18.05%, lower angular dependence, and ~ 35% lower efficiency roll-off at 1000 cd m^−2^ compared to the control TEOLEDs. These device performances are superior to those of blue OLEDs reported in recent years [3, 34, 35], which are attributed to the synergetic amalgamation of the CSE that improves the angular dependency and the MQW EML that improves the color purity and efficiency roll-off.

## Experimental Section

### OLED Fabrication

In this experiment, a 100-nm-thick Ag-coated glass substrate (AMG, Republic of Korea) was used as a reflective bottom electrode for the proposed TEOLED owing to its highly reflective and conductive properties [3]. Before device fabrication, the patterned Ag-coated glass substrates were cleaned by means of ultrasonication with isopropyl alcohol and deionized water. All materials were deposited through a shadow mask on the precleaned patterned Ag-coated glass substrates by means of thermal evaporation in a high-vacuum chamber with a base pressure of 10^−7^ to 10^−8^ mbar (Daedong High Tech, Republic of Korea). The molybdenum oxide (MoO_3_) HIL, 1,3-bis(*N*-carbazolyl) benzene (mCP) HTL, 10,10-(4,4-sulfonylbis (4,1-phenylene))bis(9,9-dimethyl-9,10-dihydroacridine) (DMAC-DPS) QW emission layer, diphenyl-bis[4-(pyridin-3-yl)phenyl]silane (DPPS) potential barrier layer (PBL), and DPPS electron transport layer (ETL) were evaporated at deposition rates of 0.1, 0.3, 0.01, and 0.1 nm s^−1^, respectively. The deposition rate of the LiF and Al electron-injection layers was 0.5 nm s^−1^. Subsequently, a 12-nm-thick Ag layer and a 65-nm-thick WO_3_ layer were deposited at a rate of 0.5 nm s^−1^ through a square shadow mask. Finally, a 12-nm-thick Ag layer and a 65-nm-thick DPPS layer were deposited through a cathode shadow mask at a rate of 0.5 nm s^−1^. The 4 mm^2^ (2 × 2 mm^2^) emission area was finely defined based on the overlap between the Ag anode and the Ag/WO_3_/Ag cathode.

### OLED Characterization

Cross-sectional images of the fabricated multilayered structures were obtained using a focused ion beam transmission electron microscope (FIB-TEM) (Model: Tecnai G2 F30) at an acceleration voltage of 300 kV. The samples were prepared using a focused ion beam (FIB, Quanta 200 3D) and processed by means of ion milling. Scanning transmission electron microscopy (STEM) images were obtained using an FEI Titan 80–300. The normal incidence and total transmittance of the demonstrated electrodes were recorded using an ultraviolet–visible (UV–Vis) spectroscope (Lambda-35, PerkinElmer). Sheet resistance was measured using a semi-automated four-point probe system (Advanced Instrument Technology, CMT-SR2000N). The surface work function of the Ag and WO_3_ thin films was measured by means of ambient pressure photoemission spectroscopy. A high-intensity deuterium (D_2_) lamp was used to generate photoelectrons from the sample surface. The surface morphology and surface roughness of the organic thin films were observed using an atomic force microscope (Park Systems, XE series) in the noncontact mode.

OLED characteristics, including the current density–voltage luminance characteristics, EL intensity, external quantum efficiencies, current efficiencies, and power efficiencies, were measured using a source meter (Keithley SMU 2400) and a spectroradiometer (CS-2000, Konica Minolta) in the OLED I–V–L Test System (M6100, McScience). Angle-dependent normalized luminance was recorded using a purpose-built rotation stage in steps of 10° at 8 V.

### Optical Simulations

Finite-difference time-domain (FDTD) optical simulations based on Maxwell’s equation were performed using an FDTD software package (Lumerical, Inc.). Periodic boundary conditions in the transverse direction (x-axis) and “perfect matching layer (PML)” boundary conditions in the longitudinal direction (y-axis) were applied in this simulation. Transmittance was calculated using a plane wave source, and the field intensity was simulated by the sum of the dipole sources in the x, y, and z directions after calculation. The electric field distribution profile was simulated in three-dimensional space. The electric and far field distributions were then observed in the frequency domain by using a power monitor. The simulation time was set to 1000 fs. The refractive indices and the coefficient indices were measured using an alpha-SE Ellipsometer (WizOptics, Korea), and the measured values were used as input parameters in the modeling process.

## Result and Discussion

### Design and Concept of Proposed TADF TEOLED Structure

**Fig. 1:**
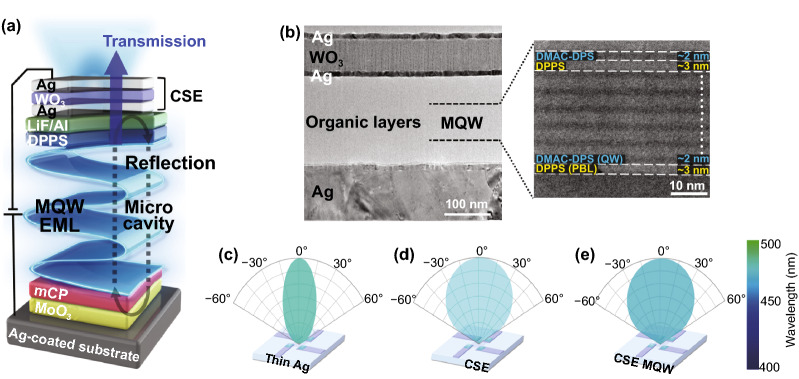
**a** Schematic of a blue TADF top emission OLED with an MQW structure and a multilayer cathode; **b** cross-sectional transmission electron microscopy (TEM) image of the proposed structure (left), and enlarged image of a QW structure (right) obtained by scanning TEM (STEM); and **c**–**e** conceptual light emission profiles depicting the advantages of the CSE-MQW device over thin Ag and CSE-only devices. (Color figure online)

Figure 1a shows a schematic of the proposed TADF TEOLED structure comprising glass/Ag/MoO_3_/mCP/MQW/DPPS/LiF/Al/CSE and fabricated on a reflective Ag-coated glass substrate. Figure 1b (left) shows a cross-sectional transmission electron microscopy (TEM) image of the TADF-TEOLED fabricated using MQW and CSE layers with optimized thicknesses. Distinct interfaces between the WO_3_ and the top and bottom Ag layers can be observed with complete surface coverage. Figure 1b (right) depicts a magnified scanning TEM (STEM) image of the seven QW structures formed through alternate deposition of 10,10-(4,4-sulfonylbis (4,1-phenylene))bis(9,9-dimethyl-9,10-dihydroacridine) (DMAC-DPS) layers (≈ 2 nm) and diphenyl-bis[4-(pyridin-3-yl)phenyl]silane (DPPS) layers (≈ 3 nm). Clearly, the EML consisting of DMAC-DPS QW and DPPS PBL in the MQW structure is well defined. Because the deposition conditions were well optimized, the alternate organic layers exhibited uniform thickness and homogenous coverage. Figure 1c–e presents the conceptual perspective of the present study. In the thin Ag cathode (Fig. 1c), high color purity can be achieved, but the distribution of emitted light is narrow because of microcavity effects. By contrast, wide light distribution can be obtained in the CSE-based devices, but color purity may not be good owing to inefficient exciton confinement (Fig. 1d). These limitations could be overcome by employing transparent CSE and MQW EML, which offer wide distribution of emitted light and good color purity (Fig. 1e).

### Optical and Electrical Properties of CSE

**Fig. 2:**
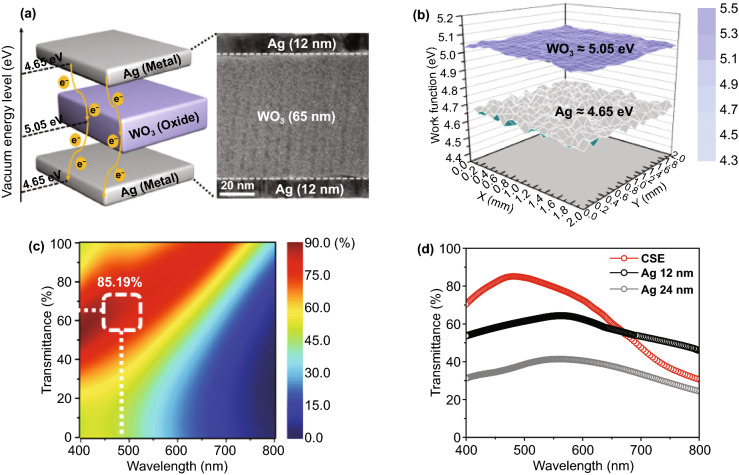
**a** Schematic of the proposed CSE along with its energy band diagram (left) and enlarged cross-sectional TEM image (right). **b** Surface work function mapping images of the Ag and WO_3_ layers. **c** Simulated transmittance contour plots of CSE structures as a function of the WO_3_ thickness between two 12-nm-thick Ag layers. **d** Measured optical transmittance of CSE, Ag (12 nm), and Ag (24 nm) covered with DPPS (*t*_DPPS_ = 65 nm) layers on a glass substrate. In this simulation and measurement, a 1 nm Al adhesion layer is included to consider the metallic effect, but a 1-nm LiF layer is not considered for simplicity because its influence on the transmittance is negligible

Figure 2a shows a schematic of electron transport in the Ag (12 nm)/WO_3_ (65 nm)/Ag (12 nm) (left) structure and a cross-sectional TEM image (right) of the CSE. Evidently, the Ag and WO_3_ layers formed heterointerfaces with complete surface coverage, which is consistent with the CSE structure in the fabricated TADF-TEOLED device (Fig. 1b). The work function of Ag (Φ = 4.65 eV) is smaller than that of WO_3_ (Φ = 5.05 eV). When these layers come into contact with each other, electrons are transported from Ag to WO_3_, leading to the alignment of Fermi levels at equilibrium. Furthermore, band bending occurs owing to the Fermi level alignment, leading to accumulation-type (ohmic) contact at the Ag/WO_3_ interface [36, 37]. In this case, electrons can flow easily from Ag to WO_3_ without any barrier. This is evident from the surface work function mapping image (Fig. 2b) as well, which was obtained using a Kelvin probe system. The work function difference between Ag (4.65 eV) and WO_3_ (5.05 eV) is 0.4 eV, which is favorable for ohmic contact among electrons between Ag and WO_3_.

Optical simulations based on Maxwell’s equations were performed to optimize the transmittance of the CSE. In the present study, we fixed the thickness of the Ag layer to 12 nm based on the simulation results (Fig. S1) to secure the continuous film properties of the Ag layer [38, 39]. Figure 2c shows contour plots of the simulated optical transmittance of the CSE structure for different WO_3_ thicknesses. The transmittance of the CSE structure is the maximum overall when the WO_3_ thickness is 65 nm. The measured maximum transmittance was 85% at the wavelength of 480 nm, which is consistent with the simulation results (Fig. S2a). Furthermore, the optical transmittance decreased at higher wavelengths, irrespective of the WO_3_ thickness. Figure 2d shows the transmittance spectra of the Ag thin films (12 and 24 nm) and the CSE covered with a DPPS (65 nm) layer on a glass substrate. Compared to the Ag thin films (12 and 24 nm), the CSE exhibited higher transmittance in the blue wavelength range, probably because of the anti-reflection effect due to destructive interference of the reflected waves generated between the double-metal and high-refractive-index oxide layers of the CSE. This MOM structure is widely used to enhance conductivity and optical transmittance [23, 27]. The influence of the ultra-thin LiF (1 nm) and Al (1 nm) layers on the transmittance of the electrode is discussed in Fig. S2c-d. The transparent CSE exhibited superior conductivity with a lower sheet resistance of 2.24 Ω sq^−1^ compared to the 12 nm Ag thin film (8.74 Ω sq^−1^) (Table S1). In addition, we computed the FoM by using Eq. (1) to investigate the trade-off relationship between the transmittance and sheet resistance of the proposed electrode.

1 $$ {\text{FoM }} = \left( {{\frac{{T^{10} }}{{R_{S} }}}} \right) \times 1000 $$

where *T* and *Rs* denote the optical transmittance and sheet resistance of the electrode, respectively.

**Fig. 3:**
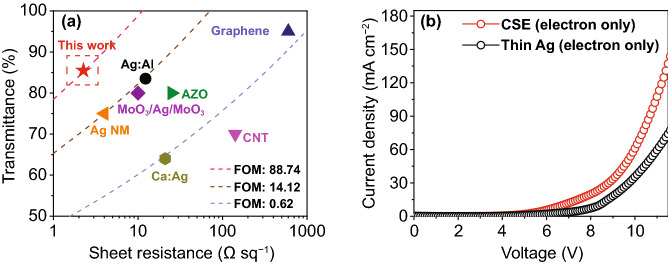
**a** Transmittance at 480 nm and sheet resistance of the Ag/WO_3_/Ag electrode in comparison with those of the previously reported transparent electrodes. The dashed lines denoting the figures of merits are provided to facilitate direct comparison. **b** *J*–*V* curves of the electron-only device with a CSE and thin Ag electrodes

Figure 3a shows the transmittance and sheet resistance of the CSE in comparison with those of the previously reported transparent electrodes [6, 40–45]. The proposed CSE has a higher FoM (88.74 10^−3^ Ω^−1^) than those of the previously reported Al-doped Ag (Ag:Al) [6], graphene [40], Al-doped zinc oxide [41], Ag nanomesh (Ag NM) [42], CNT [43], MoO_3_/Ag/MoO_3_ [44], and Ca:Ag [45]. The dashed lines indicate that the proposed CSE has a considerably higher FoM than the other transparent electrodes. The lower sheet resistance of the CSE can be attributed to the negligible longitudinal resistance of the sandwiched WO_3_, as explained in Supplementary Information [26]. Hence, CSE exhibits low sheet resistance and a well-matched work function, which is beneficial for efficient charge injection.

The charge balance behavior of the CSE and electron transport layer was investigated by fabricating two electron-only devices (EODs) with CSE and thin Ag electrode layers. The configurations of the two EODs were as follows: Ag (100 nm)/LiF (3 nm)/DMAC-DPS (36 nm)/DPPS (35 nm)/LiF/Al/CSE and Ag (100 nm)/LiF (3 nm)/DMAC-DPS (36 nm)/DPPS (35 nm)/LiF/Al/thin Ag (12 nm), respectively. A 65-nm-thick DPPS capping layer was introduced to prevent degradation of device performance due to Ag oxidation. The thickness of the capping layer was optimized based on the results of an optical simulation (Fig. S2b). The *J*–*V* characteristics (Fig. 3b) indicate that the current density of the CSE device was three to four orders of magnitude higher than that of the thin Ag device. The higher level of electron injection in the CSE device was attributed to the lower sheet resistance of CSE (2.24 Ω sq^−1^) compared to that of the thin Ag electrode (8.74 Ω sq^−1^) and balanced electron flows across the CSE.

As explained in Sect. 3.2, the Ag/WO_3_ interfacial contact is ohmic, and the potential barrier between CSE/Al/LiF and DPPS (ETL) is equivalent to that between Ag/Al/LiF and DPPS because the bottom Ag in the CSE and the thin Ag electrode have the same work function. Therefore, the current density is essentially determined by the sheet resistance in both devices [46]. Additionally, the built-in potential produced across the WO_3_ layer in the CSE helps establish a balanced electron flow, which increases the current density [21]. Notably, charge carriers are injected from both electrodes under forward biases, but the injection rate differs initially at the interface of the CSE structure, which leads to the development of a built-in potential across the WO_3_ layer. These notable properties of the CSE imply that it could serve as a potential transparent electrode in optoelectronic applications.

### Effect of EML (DMAC-DPS) Thickness on SQW TADF-TEOLED Performance

First, we fabricated TADF-TEOLEDs with bulk EML (≈ 36 nm) and a CSE as the top electrode. Even though the device emitted blue light centered at 475 nm, the emission was too broad, with a full-width half maximum (FWHM) value of approximately 85 nm, and it exhibited low color purity. Furthermore, the device suffered from severe efficiency roll-off (Table S2), mainly because of triplet aggregation in the bulk EML; the aggregated triplets were quenched by polarons (TPA) and other triplets (TTA). To reduce the efficiency roll-off due to TPA and TTA, we fabricated TEOLED devices with the MQW structure to confine the charge carriers and excitons and broaden the ERZ. This approach has been widely used to fabricate inorganic LEDs, and it is considered effective to increase the efficiency of OLEDs [30, 47].

**Fig. 4:**
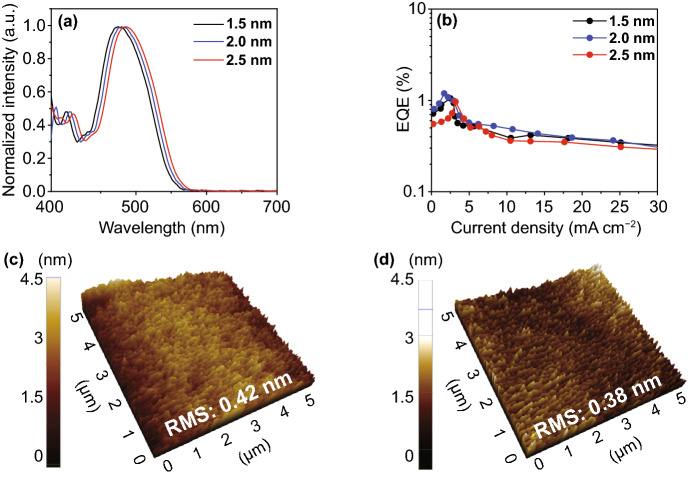
**a** Normalized EL spectra at 6 V. **b** EQE-current density curves of a single-quantum well (SQW) device. AFM images of the **c** glass/DPPS (3.15 nm)/bulk DMAC-DPS (36 nm) and **d** glass/DPPS (3.15 nm)/DMAC-DPS (2 nm) films

To optimize the performance of the proposed TADF-TEOLEDs, we fabricated three prototype devices with the following configuration: glass/Ag (100 nm)/MoO_3_ (2 nm)/mCP (125 nm)/single QW (SQW) [DPPS (36 − *x* nm)/DMAC-DPS (*x* nm)]/DPPS (35 nm)/LiF/Al/CSE/DPPS. The total EML thickness was fixed to 36 nm, and the thickness of the DMAC-DPS layer was varied as *x* = 1.5, 2.0, and 2.5 nm. Figure 4a compares the EL spectra of the TADF-TEOLEDs fabricated with DMAC-DPS layers of different thicknesses. For all DMAC-DPS layers, a peak was observed near the target wavelength of 480 nm; however, its position was shifted to a higher wavelength in case of the thicker layers. This redshift was thought to result from the quantum-size effect in organic thin films [48]. Figure 4b shows the variation in EQE with the current density of the SQW TADF-TEOLEDs fabricated with DMAC-DPS layers of different thicknesses. The measured peak EQE values of the 1.5-, 2.0-, and 2.5-nm-thick DMAC-DPS films were 1.01%, 1.2%, and 0.9%, respectively. This variation in the EQE values with the DMAC-DPS layer thickness can be explained using AFM images of the DMAC-DPS films of different thicknesses deposited on DPPS (3.15 nm), as illustrated in Figs. 4c–d and S3. The root mean square (RMS) roughness, measured by AFM, on the surface of bare glass was 0.25 nm (Fig. S3a) and that on the bulk DMAC-DPS film deposited on the glass was 0.42 nm (Fig. 4c). However, the 1.5-nm-thick DMAC-DPS film exhibited aggregations on its surface, which, coupled with nonhomogeneous interfaces, increased the RMS value to 0.88 nm (Fig. S3b), as is evident from the STEM image (Fig. S3c). The nonhomogeneous interfaces and aggregation might induce TPA and TTA owing to poor charge transport characteristics, leading to a reduction in device performance [31]. When the thickness of the DMAC-DPS film was increased to 2 nm, the RMS value of the surface decreased to 0.38 nm (Fig. 4d), which resulted in the formation of a homogenous interface, as shown in Fig. 1b. The smooth surface of the DMAC-DPS film might give rise to a homogeneous interface with DPPS PBLs when constructing QW, eventually improving device performances such as EQE, luminance, and efficiency roll-off owing to reduced charge/exciton aggregations in the QW EML. In addition, the peak EL intensity was observed at a target wavelength of 480 nm. By contrast, as the thickness of the DMAC-DPS film was increased to 2.5 nm, the EQE at the target wavelength decreased owing to a peak shift to longer wavelengths, as observed in Fig. 4a. Thus, 2 nm was selected as the optimum DMAC-DPS thickness to realize superior device performance at the target wavelength. Furthermore, to enhance the energy transfer from DPPS to DMAC-DPS and efficiently confine the ERZ excitons, MQW structures were constructed.

### Effect of the Number of QWs on Device Characteristics

**Fig. 5:**
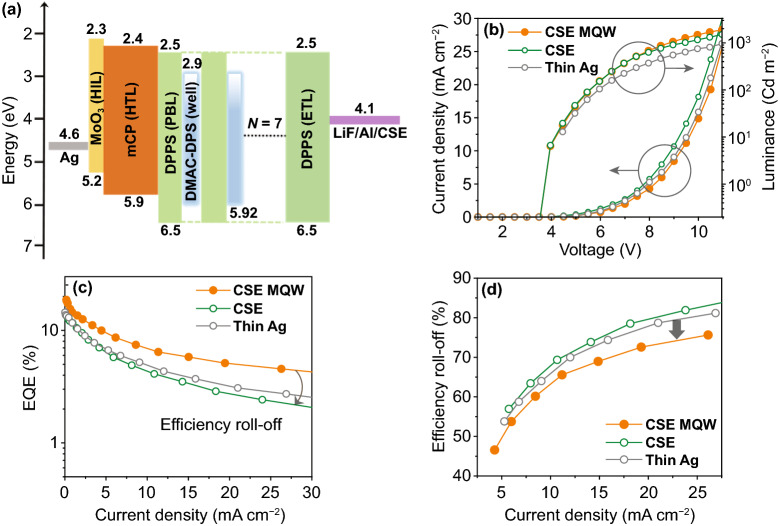
**a** Schematic and energy level diagram of the proposed TADF-TEOLED with an MQW structure (*Devices I*). **b** Current density (*J*)-voltage (*V*)-luminance (*L*) plots. **c** EQE-current density plots. **d** Efficiency roll-off versus current density plots

**Fig. 6:**
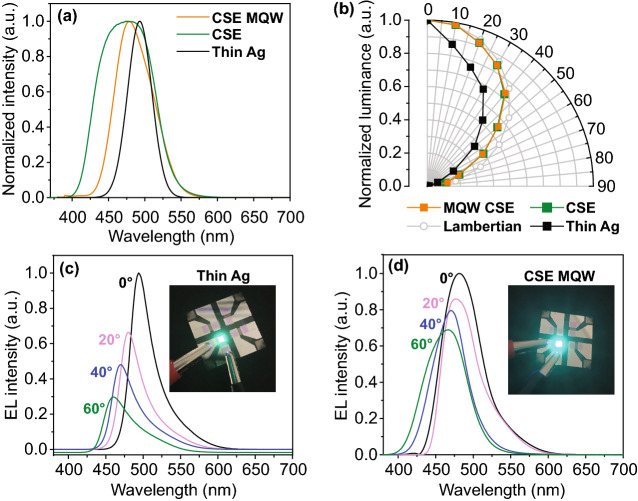
**a** Normalized EL spectra at 8 V. **b** Angle-dependent normalized luminance at 8 V (= 0.17 mA) for *Devices I*, *II*, and *III*. The luminance curves of the BEOLED with the ITO electrode measured at 8 V (= 0.12 mA) and Lambertian distributions are provided as well for comparison. EL spectra obtained at different viewing angles at 8 V for **c** *Device III* and **d** *Device I*. The insets of **c** and **d** are photographs of the light emission of *Devices III* and *I*, respectively, taken at near-zero degree

The configuration of the fabricated TADF-TEOLEDs with the CSE and MQW structures is as follows: glass/Ag (100 nm)/MoO_3_ (2 nm)/mCP (125 nm)/[DPPS/DMAC-DPS]n/DPPS (35 nm)/LiF/Al/ CSE/DPPS; the corresponding energy level diagram is shown in Fig. 5a. The performance of the devices fabricated using different numbers (*n*) of QWs is shown in Fig. S4. The EQE of the MQW devices (Fig. S4a) was higher than that of the SQW device (Fig. 4b), and the device with seven QWs exhibited a peak EQE of approximately 18.05% with low-efficiency roll-off. These results might be due to that fact that optimized MQWs not only increase the ERZ but also enhance the quantum confinement effect. In addition, well-confined charges and excitons in MQWs give rise to the higher photoluminescence (PL) quantum yield due to the reduced PL quenching [49]. However, the EQE decreased to 10.7% when the number of QWs was increased to nine. As the number of QWs is greater than the optimum number (*n* = 7), the PBL thickness decreases, leading to excess charge carrier tunneling. Accordingly, the weakened exciton confinement decreases the EQE. The devices with fewer QWs lacked interfaces for charge carrier accumulation and exhibited lower EQE and high efficiency roll-off. The EQE values presented here were measured using a spectroradiometer (CS-2000) without calibration. Figure S4b shows that all of the MQW-structured devices exhibited nearly the same turn-on voltage (Vt) (Vt: Voltage at the luminance of 0.1 Cd m^−2^). Furthermore, at a given voltage, the current density decreased as the number of QWs increased. As the number of QWs increased, a greater number of DPPS/DMAC-DPS interfaces were generated; thus, the defects or traps in the DMAC-DPS films or those at the DPPS/DMAC-DPS interfaces decreased the current density of the devices [31]. Accordingly, for nine QW devices, the current density was significantly decreased. The EL spectra of the devices with different numbers of QWs are illustrated in Fig. S4c. The EL spectrum of the device with three QWs had a peak at 480 nm and a weak shoulder peak at around 400 nm. The weak emission at 400 nm was ascribed to inefficient energy transfer between the DPPS and DMAC-DPS and exciton leakage into the mCP layer owing to insufficient interfaces for exciton confinement [31]. The weak shoulder peak disappeared in the spectra of the devices with five and seven QWs, and this disappearance was ascribed to efficient exciton confinement. In particular, devices with seven QWs showed a stable EL spectrum owing to the effective confinement of charges and excitons in the EML. For the device with nine QWs, the EL spectrum showed a broader peak (Fig. S4c), which is similar to the EL spectrum observed for the device without any MQW structure (Fig. 6a). This may be because the thickness of PBL decreases as the number of QWs increases within the fixed thickness of the EML; consequently, carrier confinement is reduced, as reported elsewhere [50].

### Device Performance of Fabricated TADF-TEOLEDs

The performance of the optimized MQW EML devices was compared with that of bulk-EML-based TEOLED devices with thin Ag cathode films and those with a CSE as the cathode to understand the synergistic effect of the MQW and CSE. The following three types of devices were fabricated:

*Device I: Ag/MoO*_*3*_*/mCP/[DPPS/DMAC-DPS]*_*7*_*/DPPS/LiF/Al/CSE/DPPS* (here, the thickness of the entire EML, including the quantum barrier, was set to 36 nm, and the thickness of the DMAC-DPS QW and the DPPS quantum barrier were set to 2 and 3.15 nm, respectively.)

*Device II: Ag/MoO*_*3*_ */mCP/DMAC-DPS/DPPS/LiF/Al/CSE/DPPS*

*Device III: Ag/MoO*_*3*_ */mCP/DMAC-DPS/DPPS/LiF/Al/Ag/DPPS*

All of these devices were covered with an organic outcoupling DPPS (65 nm) layer to passivate the top Ag layer.

Figure 5b shows the current density (*J*)-voltage (*V*)-luminescence (*L*) characteristics of the fabricated devices. All devices had a similar turn-on voltage (4.0 V). Furthermore, the current density of *Device I* was lower at a specific drive voltage, and its brightness was higher than that of *Devices II* and *III*. This enhanced luminance and EQE were attributed to the introduction of DPPS in the MQW structure. DPPS was employed as a PBL owing to its wide bandgap that can adequately confine the DMAC-DPS. In general, hole mobility is considerably greater than electron mobility in OLEDs, and for this reason, holes should be blocked effectively to maintain the charge balance [51]. As a PBL with a deep HOMO level, the DPPS can effectively block holes to maintain the charge balance. The hole mobility (≈ 1.2 × 10^−4^ cm^2^ V^−1^ s^−1^) of the mCP hole-transport layer (HTL) [52] was higher than the electron mobility (≤ 10^−6^ cm^2^ V^−1^ s^−1^) of the DPPS ETL [53], which is detrimental to device performance in conventional OLEDs. However, the lower mobility of DPPS and its hole-blocking ability promote recombination at the DPPS/DMAC-DPS interface in the MQW structure. In this manner, the recombination zone is broadened along the MQW structure with homogeneous DPPS/DMAC-DPS interfaces, resulting in enhanced luminance and EQE. To investigate the reliability of this remarkable device performance, we statistically measured the EQE, FWHM and efficiency roll-off distributions of 10 different devices and found a high level of reproducibility in terms of device performance (Fig. S5 and Table S2). *Device I* exhibited a high maximum EQE (18.05%) and suppressed efficiency roll-off at high current densities (Fig. 5c). For non-MQW devices, the aggregation of electrons and holes in the bulk EML increased the triplet population. This induced TPA and TTA, and hence, severe efficiency roll-off. By contrast, the QW structure broadened the ERZ and increased charge carrier distribution at the DPPS/DMAC-DPS interface. The uniform charge carrier distribution at the interface prevented TPA and TTA, thereby suppressing efficiency roll-off in *Device I* (Fig. 5d). Significantly, *Device I* with the CSE and MQW structures exhibited reduced efficiency roll-off over the entire range of current density tested herein, which was approximately 27% and 35% lower than those of Device II and III without the MQW structure at 1000 cd m^−2^.

Figure 6a shows the EL spectra of *Devices I*, *II*, *and III*. The spectra of *Devices I* and *II* contain emission peaks at around 480 and 475 nm, respectively, whereas the spectrum of *Device III* with an Ag thin film electrode contains an emission peak at around 493 nm. The OLED performance parameters of these devices are summarized in Table S2. Furthermore, *Devices I* and *II* exhibited a broad emission peak, unlike *Device III*. These broad EL spectra of *Devices I* and *II* (CSE-based devices) can be attributed to the suppressed microcavity effect in the CSE [54]. Although broad emission is beneficial for lighting applications, it indicates low color purity and is undesirable for display applications [46]. The lower FWHM value of *Device I* (≈ 60 nm) compared to that of *Device II* (≈85 nm) can be ascribed to the QW structure, which is consistent with previously reported OLEDs having the MQW structure [28, 31]. The broad EL spectrum of *Device II* (without MQW) can be attributed to the charge-transfer excited state properties of conventional TADF emitters. By contrast, in the case of *Device I* (with MQW), the excitons from the quantized energy states in QWs cause narrowing of the EL spectrum [55]. These results indicate that combining the QW structure with a microcavity-suppressing electrode improved the color purity of the fabricated OLEDs.

In addition to color purity, we investigated the viewing angle characteristics of the fabricated devices. Spectrum analysis was performed by conducting an optical far-field simulation. Figure S6a-b shows the variation in EL intensity at different viewing angles for the thin Ag-based TEOLEDs and the CSE/MQW-based TEOLEDs, respectively. Interestingly, it was confirmed that the proposed device maintained its intensity over a wider viewing angle range (≈ 60°) than the thin Ag-based TEOLEDs at the target wavelength of 480 nm. This indicates that the proposed structure has lower angular dependence. Therefore, we fabricated bulk-EML-based TEOLEDs by using a thin Ag cathode and MQW EML-based TEOLEDs by using a CSE to investigate various device properties, including color shift characteristics and angle-dependent emission intensity distributions. In general, because constructive and destructive interference are affected by the cavity length of the TEOLEDs employing the microcavity effect, the luminance decrease and color difference are severe because of variations in the cavity length with the viewing angle. Accordingly, the angle-dependent emission intensity distributions of the Ag-based device with the microcavity effect and the CSE-based device with suppressed cavity effect are remarkably different, as shown in Fig. 6b. The luminance curve of the Lambertian distribution is provided for comparison. The angular emission distribution of the CSE-based devices is close to the Lambertian curve, which is usually observed in the case of TEOLEDs with a transparent electrode. By contrast, the emission distribution of the thin Ag-based device is deviated from the Lambertian pattern. Similar results were observed for the measured and calibrated EQE values of *Devices I* and *III* (Fig. S7); that is, the measured EQE of *Device I*, which closely approximates the Lambertian curve, was similar to the calibrated EQE, whereas significant differences were observed in the measured and calibrated EQEs for *Device III*. Here, the EQE calibration was conducted using a mean estimation method under different viewing angles [56]. Moreover, the CSE TEOLED exhibited a broad and stable light extraction profile without the surface plasmon polariton (SPP) mode (Fig. S8a), whereas the Ag-TEOLED exhibited a narrow light extraction profile owing to micro-cavity effects coupled with the field distribution of the SPP mode (Fig. S8b). This result is consistent with the calculated light extraction efficiency (Fig. S8c). Although the Ag-based TEOLED with the micro-cavity effect exhibited increased light extraction, its light extraction efficiency was lower than that of the CSE TEOLED because of optical losses related to the SPP mode at the interface and internal reflections [57]. Furthermore, the color shift value of the optimized CSE/MQW-based device was approximately 14 nm (Fig. 6c) lower than that of the device fabricated with an Ag thin film (≈ 36 nm, Fig. 6d). The proposed structure not only effectively suppresses the microcavity effect but also prevents luminance degradation and viewing-angle-dependent color variation. In particular, in case of the Ag-based device, the magnitude of EL intensity decreased significantly as the viewing angle increased, whereas in case of the CSE/MQW-based device, the magnitude of EL intensity varied marginally. These results are consistent with our conceptual perception (Fig. 1), as mentioned in Sect. 3.1. In previous studies, weak light-matter coupling was achieved using complex molecule structures or nanoporous polymer films, which involved complex processing techniques [13, 16, 46]. The design and use of a cavity-suppressed transparent cathode in blue TADF-TEOLEDs is described in this study, and it involves the use of facile fabrication techniques. Table S3 compares the performance of the proposed TEOLED with that of previously reported blue top and bottom emission OLEDs. Evidently, the previous studies focused either on cavity effect to achieve good angular characteristics or on the material to achieve superior color purity and efficiency roll-off. However, we focused on improving the angular characteristics, color purity, and efficiency simultaneously. Furthermore, the fabricated CSE/MQW TEOLEDs exhibited superior performance than the previously reported TEOLEDs in all aspects. This is attributed to the synergetic amalgamation of the CSE that contributed to improved viewing angle characteristics and to the MQW EML that contributed to the improved color purity and the efficiency roll-off structure, all of which led to remarkable device performance. In sum, in this paper, we described a framework for achieving a trade-off between the required parameters to potentially improve the viewing angle characteristics and color purity, as well as efficiency roll-off issues, simultaneously.

## Conclusion

A highly efficient multifunctional blue TADF-TEOLED was fabricated by combining a transparent cavity-suppressing electrode and an MQW-structured EML for the first time. The simulation and experimental results revealed that the thickness of WO_3_ (spacer) in the CSE, thickness of the EML, and number of QWs in the MQW structure strongly influence the performance of the devices fabricated herein. The multilayered structure of the CSE was optimized based on the results of an optical simulation. The device based on the optimized structure exhibited a high transmittance of approximately 85% at the target wavelength of 480 nm and a low sheet resistance of 2.24 Ω sq^−1^. Despite its higher EQE and improved angular characteristics, the CSE-based TADF-TEOLED suffered from low color purity and severe efficiency roll-off. In this study, these limitations were alleviated by employing a quantum well-structured EML. The thickness and number of QWs were optimized experimentally. The blue TADF-TEOLED comprising an CSE as the top cathode and an EML with seven QWs had a higher EQE (18.05%) than that of the control devices without any degradation in color purity, efficiency roll-off, and angular dependency. The improved device performance is attributed to the synergistic effect of the CSE and MQW-based EML. The CSE minimized the reflection of emitted light, which suppressed the microcavity effect and angular dependence in TEOLEDs. Moreover, the MQW structure contributed to the efficient confinement of charge carriers and excitons, which improved the color purity and reduced the efficiency roll-off of the devices. Significantly, the TADF-TEOLED consisting of a CSE and the MQW structure exhibited much lower efficiency roll-off over the entire range of current density compared to the devices without the CSE and MQW structure. Thus, the proposed device scheme offers a simple but practical approach for optimizing multiple output characteristics of OLEDs.

## Supplementary Information

Below is the link to the electronic supplementary material.

Supplementary file1 (PDF 1028 kb)
