# Supplementary material for: Cavity-Suppressing Electrode Integrated with Multi-Quantum Well Emitter: A Universal Approach Toward High-Performance Blue TADF Top Emission OLED
Source: Nanomicro Lett. 2022 Feb 11;14:60. doi: 10.1007/s40820-022-00802-y (PMC8837740; doi:10.1007/s40820-022-00802-y)
Supplement: Supplementary file 1 — Supplementary file1 (PDF 1028 kb) [file 40820_2022_802_MOESM1_ESM.pdf]

Supporting Information for

Cavity-Suppressing Electrode Integrated with Multi-Quantum-Well Emitter: A Universal Approach toward High-Performance Blue TADF Top-Emission OLED

Il Gyu Jang[1], Dr. V. Murugadoss[1], Tae Hoon Park[1], Kyung Rock Son[1], Ho Jin Lee[1], WanQi Ren[1], Min Ji Yu[1], and Tae Geun Kim[1,] *

[1]School of Electrical Engineering, Korea University, Anam-ro 145, Seongbuk-gu, Seoul 02841, Republic of Korea

*Corresponding author. E-mail: tgkim1@korea.ac.kr (Tae Geun Kim)

S1 Equivalent Sheet Resistance of Ag/WO3/Ag CSE

The lower sheet resistance of the metal/oxide/metal (MOM) structure can be explained using the following equation.

$$R_s = \left(\frac{1}{R_{Ag1}} + \frac{1}{R_{Ag2}}\right)^{-1} + \left(\frac{R_{Ag1}\rho_{WO_3}}{(1+R_{Ag1}/R_{Ag2})^3}\right)^{\frac{1}{2}} \frac{t_d^{\frac{1}{2}}}{L}$$

Here, R_s is the sheet resistance of the CSE, R_{Ag1} is the resistance of the first Ag layer, R_{Ag2} is the resistance of the second Ag layer, ρ_{WO_3} is the resistivity of WO_3, t_d is the thickness of the WO_3 layer, and L is the electrode length. The second term arises from the longitudinal resistance of WO_3. The value of this term is negligible because L (a few tens of millimeters) is considerably greater than t_d (a few tens of nanometers). When the second term is neglected, the sheet resistance of the CSE is represented by the first term, which is equal to the effective resistance of the two Ag layers connected in parallel.

S2 Supplementary Figures and Tables

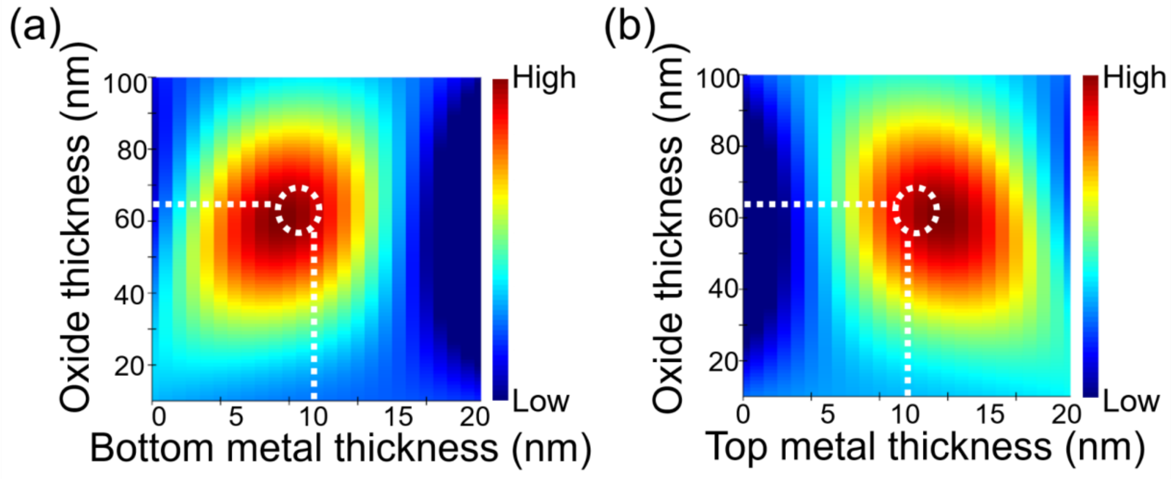

Fig. S1 Calculated optical transmittance of CSE as a function of the oxide (WO_3), top metal (Ag), and bottom metal (Ag) thickness

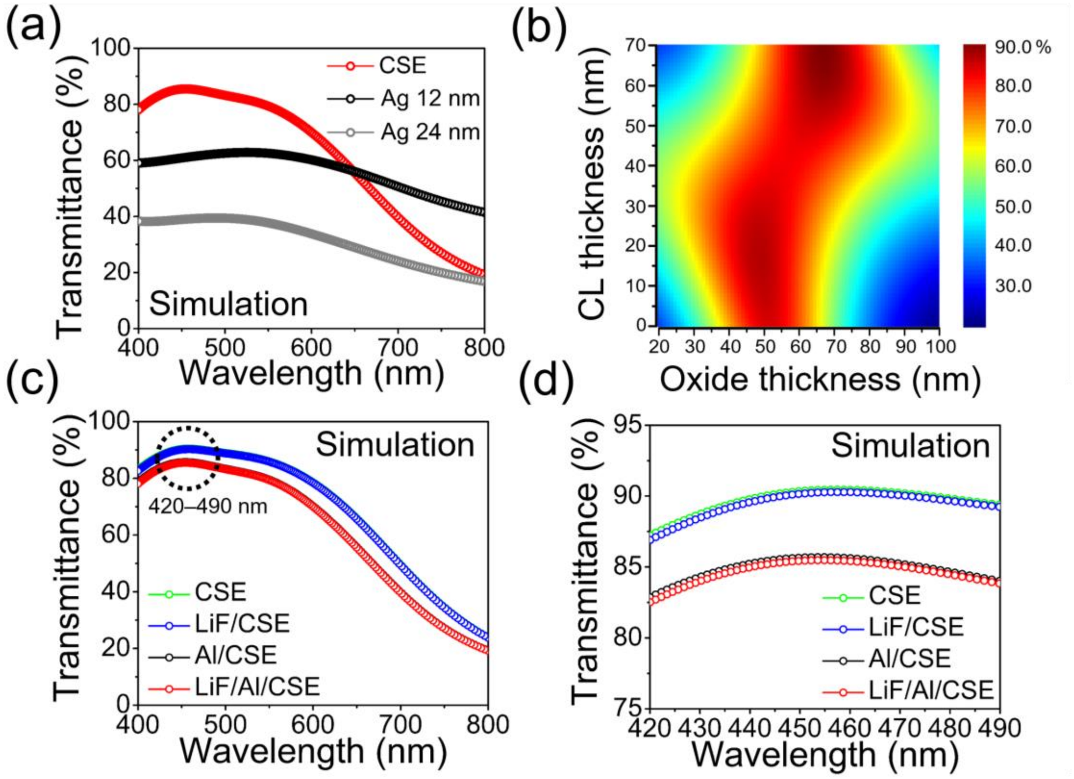

Fig. S2 **a** Transmittances of CSE and Ag thin films of thicknesses 12 and 24 nm, respectively, covered with DPPS (t_{DPPS} = 65 nm), as obtained from an optical simulation. **b** Transmittance of CSE as a function of the oxide thickness (WO_3) and capping layer thickness (DPPS). In this calculation, a 1 nm Al adhesion layer was included. **c** Calculated transmittance curves of the CSE with LiF (1 nm), Al (1 nm), and LiF (1 nm)/Al (1 nm) layers. **d** Magnified curves of (c) in the blue wavelength region (420–490 nm)

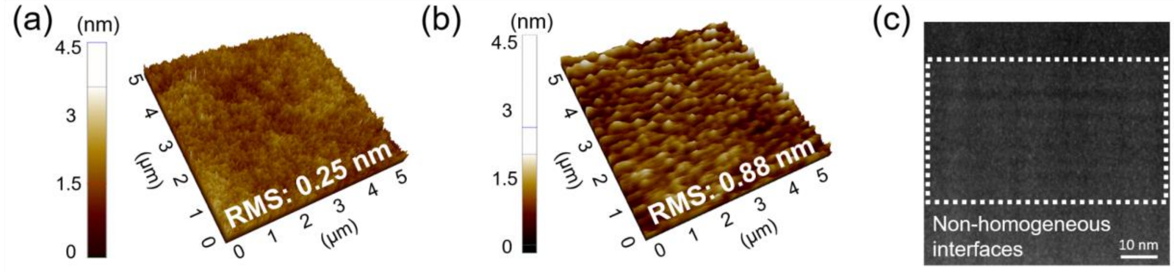

Fig. S3 AFM images of multilayer films of **a** glass and **b** glass/DPPS (3.15 nm)/DMAC-DPS (1.5 nm). **c** STEM cross-sectional image of the quantum well structure with 1.5-nm-thick DMAC-DPS and 3.15-nm-thick DPPS

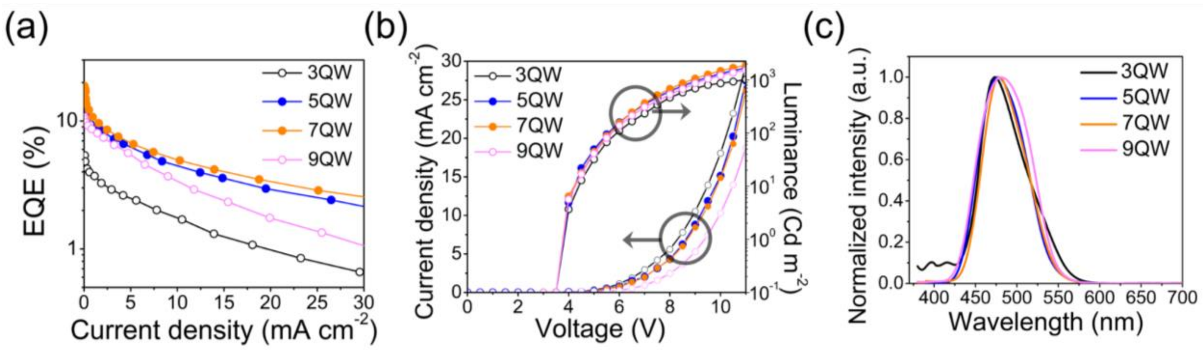

Fig. S4 **a** EQE-current density plots, **b** current density (*J*)-voltage (*V*)-luminance (*L*) plots, and **c** EL spectra at 8 V of MQW devices as a function of the number of quantum wells (*n*)

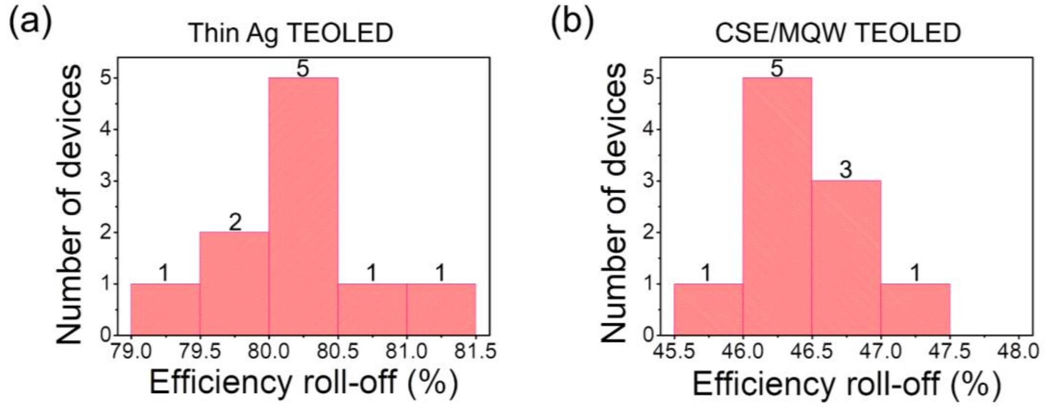

Fig. S5 Histograms of efficiency roll-off values measured @1000 cd m^{-2} from 10 different specimens of (**a**) thin Ag TEOLED and (**b**) CSE/MQW TEOLED. The values were statistically analyzed by using a standard deviation function (STDEV), and found to be in the range of 80.31 ± 0.54% and 46.48 ± 0.38%, respectively

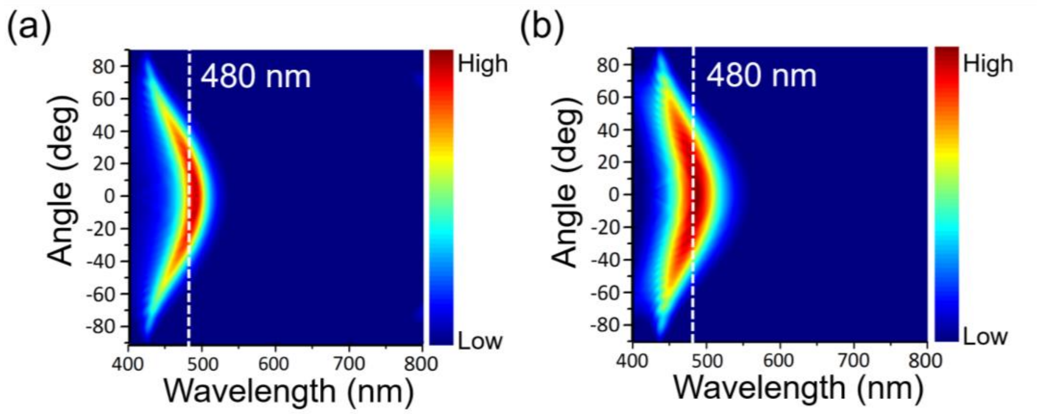

Fig. S6 Simulated far-field intensity distributions of **a** *Device III* and **b** *Device I* at different viewing angles

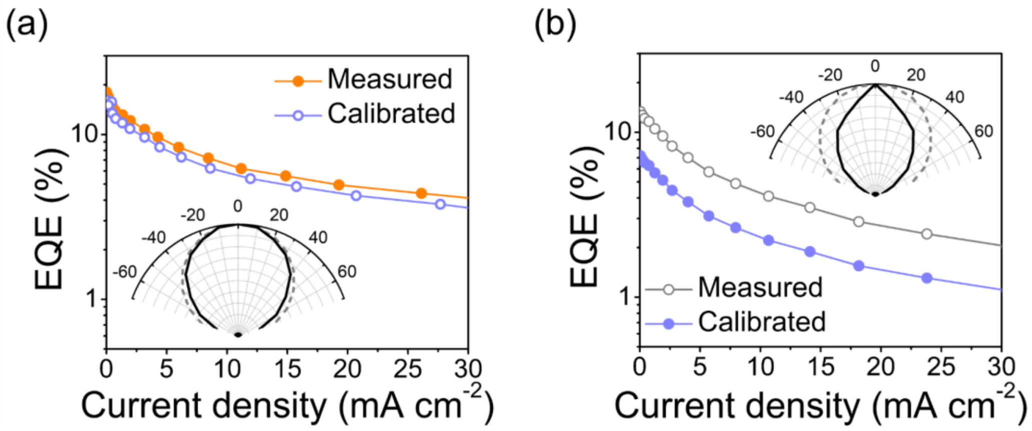

Fig. S7 Measured and calibrated EQE–current density plots of (a) Device I and (b) Device III

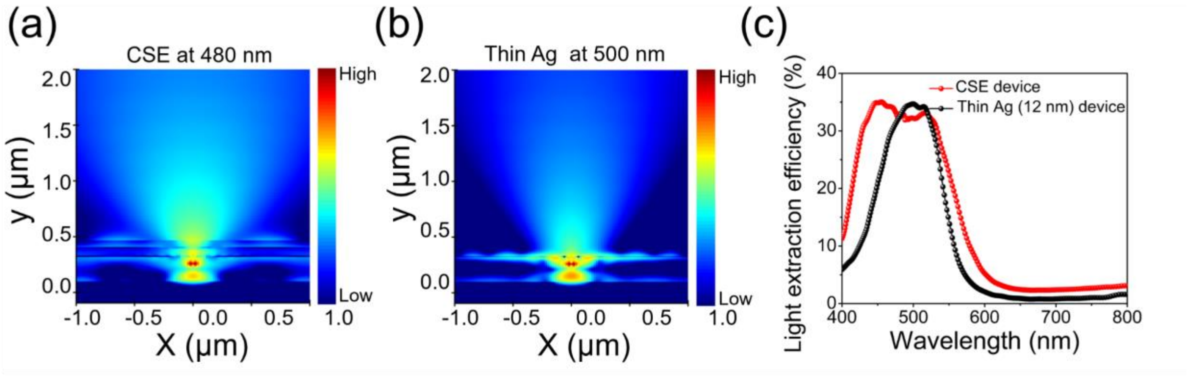

Fig. S8 Light output power distributions calculated at resonance wavelengths of **a** CSE- based TEOLED and **b** Ag-based TEOLED. **c** Calculated light extraction efficiencies of CSE- and Ag-based TEOLEDs in the wavelength region of 400–780 nm

Table S1 Characteristics of the Ag thin film electrode and transparent cavity-suppressing AWA (65 nm) electrode

Electrodes	R_s [Ω sq^{-1}]	$T_{480\ nm}$ [%]	#FoM [10^{-3} $Ω^{-1}$]
**CSE**	**2.24**	**85.1**	**88.74**
Thin Ag (12 nm)	8.74	59.4	0.62
Thin Ag (24 nm)	2.31	26.4	N/A

#Figure of merit (FoM) = $(T^{10}/R_s) \times 1000$

Table S2 Electronic properties of *Devices I*, *II*, and *III*

Device	EQE_{max} (%)	EQE_{avg} (%)	$Luminance_{max}$ (cd m^{-2})	EL peak (nm)	$FWHM_{avg}$ (nm)	Maximum efficiency roll-off @1000 cd m^{-2}
**AWA CSE/MQW *(Device I)***	**18.05**	**17.41** ± **0.45**	**2429**	**480**	**59.8** ± 0.63	**≈ 46%**
CSE (12/65/12) *(Device II)*	13.37	13.01 ± 0.29	1951	475	83.8 ± 0.93	≈ 73%
Thin Ag (12) *(Device III)*	11.39	10.83 ± 0.28	1253	493	40.4 ± 0.52	≈ 80%

Table S3 Comparison in major performances of the proposed device and previously reported blue top- and bottom-emission OLEDs

Device configuration	Max. EQE	FWHM	Angular shift	Efficiency roll-off @1000 cd m^{-2}	Refs.
Ag/Spiro-TTB: 4 wt.% F6-TCNNQ/NPB/MADN: 1.5 wt.% TBPe/BAlq/BPhen: 3 wt.% Cs/Ag/NPB (Top emission)	2.21% @460 nm	≈ 60 nm	N/A	N/A	[S1]
ITO/Ag/ITO/HATCN/BPBPA/2,6-tBumCPy/2,6-tBumCPy:46 wt.% mCBP:46 wt.%Pt(dmpzpyOczpy)	10.2% @457 nm	≈ 30 nm	N/A	N/A	[S2]

/2,7-mCpy/TmPyPB/Liq/Mg:10 wt.% Ag/CPL (Top emission)					
Ag/MoO_3/mCP/DMAC-DPS/DPEPO/LiF/Al/Ag/DPEPO (Top emission)	8.2% @472 nm	45 nm	N/A	N/A	[S3]
ITO/PEDOT:PSS/POBPCz:12 wt.% 2CzPN/TmPyPB/LiF/Al (Bottom emission)	12.9% @496 nm	≈ 90 nm	N/A	≈ 80%	[S4]
ITO/MoO_3/mCP/DPEPO:24 wt.% mSOAD/DPEPO/TPBi/LiF/Al (Bottom emission)	14.8% @480 nm	≈ 75 nm	N/A	≈ 50%	[S5]
ITO/TAPC/mCP/mCP-t-Bu:DMAC-DPS/DPPS/LiF/Al (Bottom emission)	13.5% @475 nm	≈ 90 nm	N/A	N/A	[S6]
Ag/MoO_3/mCP/DMAC-DPS/DPPS/LiF/Al/Ag/DPPS	11.39% @493 nm	41 nm	36 nm	≈ 80%	This work
**Ag/MoO_3/mCP/[DPPS/DMAC-DPS]$_7$/DPPS/LiF/Al/CSE/DPPS**	**18.05% @480 nm**	**59 nm**	**14 nm**	**≈ 46%**	**This work**

Supplementary References

[S1] Y. Deng, C. Keum, S. Hillebrandt, C. Murawski, M.C. Gather, Improving the thermal stability of top-emitting organic light-emitting diodes by modification of the anode interface. Adv. Opt. Mater. **9**(14), 2001642 (2021). https://doi.org/10.1002/adom.202001642

[S2] Y. Chen, C. Qian, K. Qin, H. Li, X. Shi et al., Ultrapure blue phosphorescent organic light-emitting diodes employing a twisted Pt(II) complex. ACS Appl. Mater. Interfaces **13**(44), 52833–52839 (2021). https://doi.org/10.1021/acsami.1c13843

[S3] W. Ren, K.R. Son, T.H. Park, V. Murugadoss, T.G. Kim, Manipulation of blue TADF top-emission OLEDs by the first-order optical cavity design: toward a highly pure blue emission and balanced charge transport. Photon. Res. **9**(8), 1502-1512 (2021). https://doi.org/10.1364/PRJ.432042

[S4] W. Li, J. Zhao, L. Li, X. Du, C. Fan et al., Efficient solution-processed blue and white OLEDs based on a high-triplet bipolar host and a blue TADF emitter. Org. Electron. **58**, 276–282 (2018). https://doi.org/10.1016/j.orgel.2018.04.027

[S5] J. Li, R. Zhang, Z. Wang, B. Zhao, J. Xie et al., Zig-zag acridine/sulfone derivative with aggregation-induced emission and enhanced thermally activated delayed fluorescence in amorphous phase for highly efficient nondoped blue organic light-emitting diodes. Adv. Opt. Mater. **6**(6), 1701256 (2018). https://doi.org/10.1002/adom.201701256

[S6] J. Keruckas, D. Volyniuk, J. Simokaitiene, E. Narbutaitis, A. Lazauskas et al., Methoxy- and tert-butyl-substituted meta-bis(N-carbazolyl)phenylenes as hosts for organic light-emitting diodes. Org. Electron, **73**, 317–326 (2019). https://doi.org/10.1016/j.orgel.2019.06.026
